# Impacts of short-term air pollution exposure on appendicitis admissions: Evidence from one of the most polluted cities in mainland China

**DOI:** 10.3389/fpubh.2023.1144310

**Published:** 2023-03-16

**Authors:** Yanhu Ji, Xuefeng Su, Fengying Zhang, Zepeng Huang, Xiaowei Zhang, Yueliang Chen, Ziyi Song, Liping Li

**Affiliations:** ^1^School of Public Health, Shantou University, Shantou, China; ^2^Injury Prevention Research Center, Shantou University Medical College, Shantou, China; ^3^Linfen People's Hospital, Linfen, China; ^4^China National Environmental Monitoring Center, Beijing, China; ^5^The Second Affiliated Hospital of Shantou University Medical College, Shantou, China

**Keywords:** short-term exposure, air pollution, appendicitis, hospital admissions, time series analysis

## Abstract

**Background:**

Emerging evidence indicates that air pollutants contribute to the development and progression of gastrointestinal diseases. However, there is scarce evidence of an association with appendicitis in mainland China.

**Methods:**

In this study, Linfen city, one of the most polluted cities in mainland China, was selected as the study site to explore whether air pollutants could affect appendicitis admissions and to identify susceptible populations. Daily data on appendicitis admissions and three principal air pollutants, including inhalable particulate matter (PM_10_), nitrogen dioxide (NO_2_), and sulfur dioxide (SO_2_) were collected in Linfen, China. The impacts of air pollutants on appendicitis were studied by using a generalized additive model (GAM) combined with the quasi-Poisson function. Stratified analyses were also performed by sex, age, and season.

**Results:**

We observed a positive association between air pollution and appendicitis admissions. For a 10 μg/m^3^ increase in pollutants at lag01, the corresponding relative risks (RRs) and 95% confidence intervals (95% CIs) were 1.0179 (1.0129–1.0230) for PM_10_, 1.0236 (1.0184–1.0288) for SO_2_, and 1.0979 (1.0704–1.1262) for NO_2_. Males and people aged 21–39 years were more susceptible to air pollutants. Regarding seasons, the effects seemed to be stronger during the cold season, but there was no statistically significant difference between the seasonal groups.

**Conclusions:**

Our findings indicated that short-term air pollution exposure was significantly correlated with appendicitis admissions, and active air pollution interventions should be implemented to reduce appendicitis hospitalizations, especially for males and people aged 21–39 years.

## Introduction

Appendicitis is an inflammation caused by a blockage of the cavity of the appendix tube for various reasons or a secondary bacterial infection (1, 2). Currently, the standard treatment for appendicitis is appendectomy, but the incidence of complications among patients is 5–28% (3, 4). In the twenty-first century, the pooled incidence of appendicitis worldwide ranges from 100 to 151 cases per 100 thousand person-years (5). In the United States, 1 in 15 people suffers from appendicitis, and appendicitis-related hospitalizations cost an average of $3 billion a year (6, 7). In China, appendicitis was one of the top five most economically burdensome diseases in 2013 (8), and the incidence of this condition has increased (7). Given the increasing incidence and financial burden of appendicitis, identifying the risk factors associated with this illness is of great importance.

Air pollution seriously affects human health and constitutes a serious global public health problem. It has been reported that 90% of the global population lives in areas where air pollution levels exceed World Health Organization (WHO) guidelines, causing ~7 million deaths each year (9). A substantial number of epidemiological studies have reported that air pollution exposure is correlated with mortality and cardiovascular, respiratory and psychiatric diseases (10, 11), but few studies have examined its relationship with appendicitis. Experimental studies have shown that air pollutants can change intestinal immunity, increase intestinal permeability and affect intestinal microbial composition (12–14), which may be related to the occurrence and development of appendicitis. Moreover, the associations observed between air pollutants and appendicitis admissions have been inconsistent in published studies. A case-crossover study conducted by Kaplan et al. in Calgary, Canada, reported that exposure to ozone (O_3_) and NO_2_ in summer was the primary risk factor for appendicitis admission (15). Subsequently, Kaplan et al. conducted a survey in 12 Canadian cities and found that the daily average maximum O_3_ level was significantly associated with perforated appendicitis admissions (16). In Taiwan, adverse effects of air pollutants (O_3_, NO_2_, and PM_10_) on daily appendicitis hospitalizations were also observed on cool days (17). However, other studies found no relationship with appendicitis admissions (18, 19). Therefore, it is necessary to conduct more studies in different regions to further clarify the association between air pollutants and appendicitis admissions.

Moreover, although the impacts of air pollutants on appendicitis have attracted increasing attention (15–18, 20), no such studies have been conducted in mainland China. With the intensification of vehicle exhaust emissions and rapid urbanization and industrialization, most of China's inland cities have faced serious air pollution situations. In addition, the concentration and composition of air pollutants vary considerably between different countries and regions (10, 21, 22), and the results of studies on the adverse effects of pollutants on appendicitis may not have been fully understood. In this study, Linfen city, a heavily polluted city in mainland China, was selected to examine the impacts of air pollutants on appendicitis admissions and to identify susceptible populations.

## Materials and methods

### Study area

**Figure 1:**
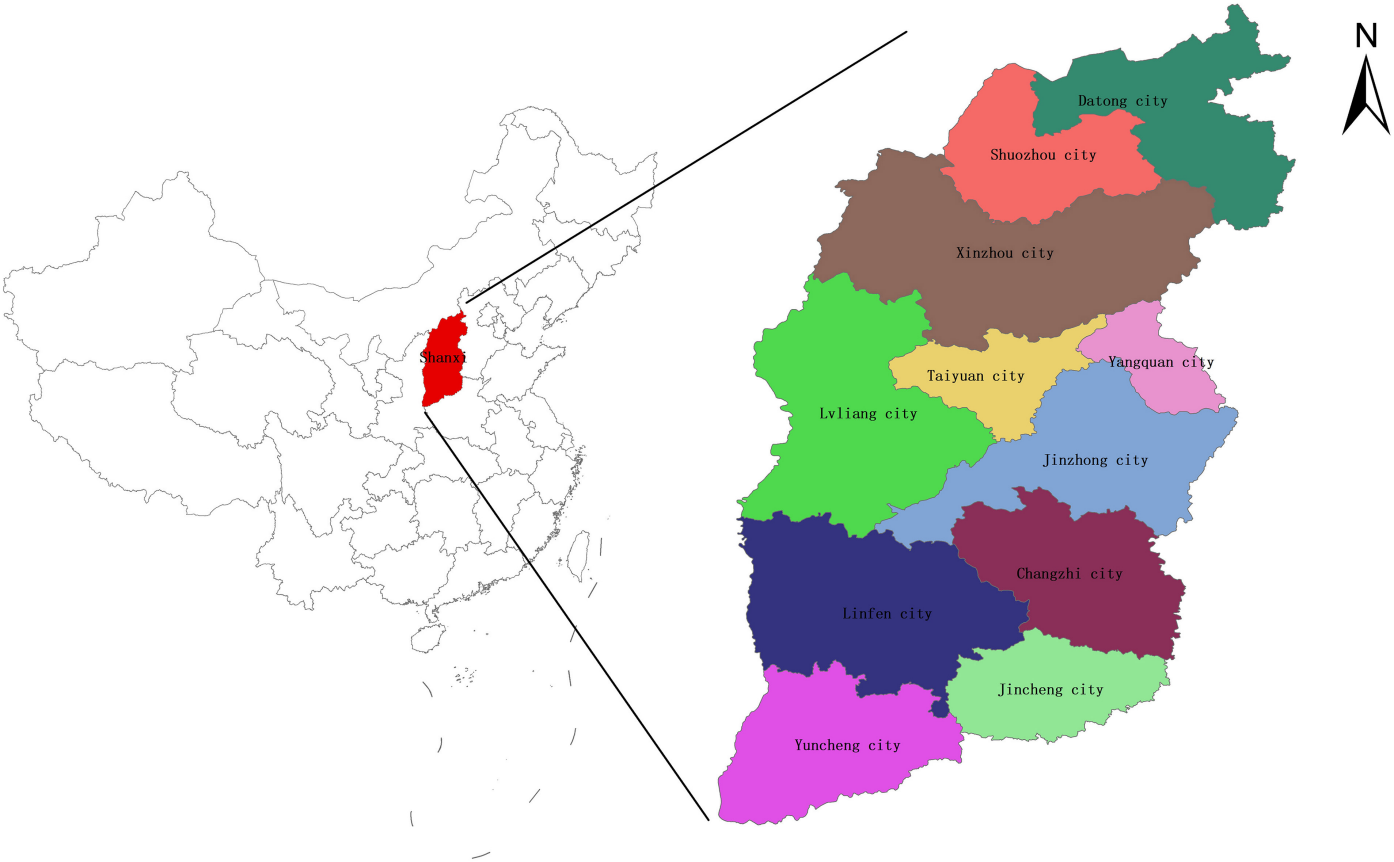
Geographical location of Linfen.

Linfen (35°23′~36°57′ N, 110°22′~112°34′ E) is in southwestern Shanxi Province, China, and has a temperate continental climate (Figure 1). By 2021, Linfen city included 1 municipal district, 14 county seats and 2 county-level cities, with a total area of 20,302 square kilometers. According to national air quality data from December 2018 and January–December 2018 released by the Ministry of Ecology and Environment of China, Linfen ranked last among 169 key cities in China, making it the city with the worst air quality in China (https://www.mee.gov.cn/). Linfen's severe air pollution comes primarily from coal mining, vehicle emissions and industrial pollution (23). In addition, Linfen is in a basin surrounded by mountains, where pollutants gather above the city, and special topographical features further exacerbate its air pollution levels.

### Data collection

Data on daily appendicitis admissions were collected from Linfen People's Hospital, which has 1,800 beds and performs an average of nearly 44,000 operations a year. As the largest comprehensive grade A hospital in Linfen city, it attracts the most patients with appendicitis to be hospitalized for treatment. We collected records from the hospital information system for all appendicitis admissions from January 1, 2016 to December 31, 2018. Data variables collected included date of admission, sex, age and home address. Appendicitis was coded and classified by experienced professionals according to the 10th revision of the International Classification of Diseases (ICD-10 codes K35.9, K35.0, and K35.1) (15). Cases with ICD-10 codes of unspecified, chronic and recurrent appendicitis were all excluded. Since air quality monitoring stations are only located in urban areas, we further excluded appendicitis cases of people who lived outside the urban area of Linfen based on their home addresses.

Daily air pollution concentrations (NO_2_, SO_2_, and PM_10_) during the same period were gathered from the Linfen Ecological Environment Bureau, which has seven national air quality monitoring stations. Daily averages from the seven monitoring stations were employed as a proxy for general air pollution levels. Meteorological data, including daily average temperature and relative humidity, were provided by the China Meteorological Data Service Network.

### Statistical analysis

We summarized the daily appendicitis hospitalizations and environmental variable data by date to form a time series dataset. Data characteristics are described as the means ± standard deviations (SDs) and quartiles. The correlation coefficients between environmental variables were estimated by Spearman correlation analysis.

The data on daily appendicitis admissions were qualitative data, and the pattern generally followed an overdispersed Poisson distribution (24). Therefore, a generalized additive model (GAM) with the quasi-Poisson function was used to evaluate the impacts of ambient air pollution on appendicitis admissions. Based on previously published studies and the minimum Akaike information criterion, a natural spline (ns) function with 7 degrees of freedom (*df)* per year was used to adjust for the long-term and seasonal trends of calendar days (25, 26). The potential confounding impacts of mean temperature (MT) and relative humidity (RH) were both adjusted by 3 *df* (27, 28). The day of the week effect (categorical variable) and holiday effect (dummy variable) were also adjusted for in the basic model. The fitted final model is as follows (Equation 1):


$$\begin{array}{l} LogE\left(Yt\right)=\beta Zt+ns\left(time,df\right)+ns\left(MT,3\right)+ns\left(RH,3\right)\\ +DOW+holiday+\text{intercept} \end{array}$$

The variables are explained as follows:

1. *t* is the observation day;
2. E(Yt) indicates the expected daily appendicitis admissions on day t;
3. β represents the log-relative rate of the exposure-response relationship;
4. Zt is the air pollutant concentration at day t.

After a core model that contained all the adjusted variables was created, air pollutants were added to the model separately. Studies have shown that the cumulative effect in the single-pollutant model may be underestimated. Therefore, we not only evaluated the single-day lag of lag0-lag5 but also applied the multiday moving average lag of lag01-lag05 to investigate the impacts of pollutants on appendicitis admissions (27, 29). Further stratified analyses were performed to investigate the modification effects of sex, age ( ≤ 20, 21–39, and ≥40) (16) and season (warm and cold) (11). Differences between groups were examined by the following formula (30) (Equation 2).


$$\begin{array}{l} \left(Q1-Q2\right)\pm 1.96\sqrt{SE1^{2}+SE2^{2}} \end{array}$$

In the formula, Q_1_ and Q_2_ are the estimates of the subgroups. SE_1_ and SE_2_ represent their respective standard errors. For example, when we performed a sex-stratified analysis, Q_1_ and SE_1_ indicated the estimated values and standard errors for males, respectively, while Q_2_ and SE_2_ were the corresponding values for females.

The robustness of the model was tested using several sensitivity analyses. First, we constructed two- and three-pollutant models to assess the confounding effects. Second, we varied the 6–9 *df* for temporal trends. Third, we also changed the *df* (3–5) for the two meteorological factors.

The R software (4.2.1) was used for all statistical analyses in this study. When the contaminant concentration increased by 10 μg/m^3^, the corresponding RR and 95% CI of appendicitis hospitalizations were expressed as the results.

## Results

Table 1 summarizes the descriptive characteristics of appendicitis admissions and environmental variables. In this study, 1,427 hospitalizations for appendicitis were included. Among these cases, 82.9% (1,183 cases) were males and 77.8% (1,110 cases) were 21–39 years old. Regarding air pollutants, the daily average concentrations were 117.42 μg/m^3^ (ranging from 16 to 658 μg/m^3^) for PM_10_, 67.62 μg/m^3^ (ranging from 4 to 858 μg/m^3^) for SO_2_ and 36.04 μg/m^3^ (ranging from 6 to 124 μg/m^3^) for NO_2_. Additionally, the daily average temperature and relative humidity were 14.87°C and 52.58%, respectively. The time series plots of pollutants are displayed in Supplementary Figure 1. The concentrations of air pollutants reached their peak in winter but showed a yearly downward trend.

**Table 1:** Basic description of appendicitis admissions, pollutant concentrations and meteorological factors in Linfen, China, 2016–2018.

**Variables**	**Sum**	**Mean (SD)**	**Min**	**P10**	**P25**	**P75**	**P90**	**Max**
Appendicitis cases	1,427	1.30 (0.95)	0	0	1	2	3	5
Male	1,183	1.08 (0.96)	0	0	0	2	2	5
Female	244	0.22 (0.47)	0	0	0	0	1	3
≤ 20 years	174	0.15 (0.38)	0	0	0	0	1	2
21–39 years	1,110	1.01 (0.98)	0	0	0	2	2	5
≥40 years	143	0.13 (0.35)	0	0	0	0	1	3
Warm season (April to September)	492	0.45 (0.65)	0	0	0	1	1	4
Cold season (October to March)	935	0.85 (1.11)	0	0	0	2	2	5
PM_10_ (μg/m^3^)	-	117.42 (77.82)	16	49.5	68	138	208	658
SO_2_ (μg/m^3^)	-	67.62 (95.21)	4	13	19	67	154	858
NO_2_ (μg/m^3^)	-	36.04 (18.38)	6	16	23	45	62	124
Mean temperature (°C)	-	14.87 (10.50)	−10.0	0.3	5.6	24.0	28.6	33.3
Relative humidity (%)	-	52.58 (17.79)	10.0	29.0	39.0	65.0	77.0	98.0

SD, standard deviation; P_10_, P_25_, P_75_, P_90_, the10th, 25th, 75th, 90th percentile; PM_10_, Particulate matter with aerodynamic diameter ≤ 10 μm; NO_2_, Nitrogen dioxide; SO_2_; Sulfur dioxide.

The Spearman correlation coefficients of the environmental variables are shown in Table 2. These pollutants were strongly correlated with one another, including PM_10_ and SO_2_ (rs = 0.66, *P* < 0.001), PM_10_ and NO_2_ (rs = 0.65, *P* < 0.001), and SO_2_ and NO_2_ (rs = 0.66, *P* < 0.001). Meteorological factors, including the mean temperature and relative humidity were negatively correlated with pollutants. However, there was a weak positive correlation between the two meteorological factors (*P* < 0.001).

**Table 2:** Spearman correlation analysis of environmental variables in Linfen, China, from 2016 to 2018.

	**PM_10_**	**SO_2_**	**NO_2_**	**Mean temperature**
SO_2_	0.66^***^			
NO_2_	0.65^***^	0.66^***^		
Mean temperature	−0.42^***^	−0.53^***^	−0.54^***^	
Relative humidity	−0.093^**^	−0.23^***^	−0.036	0.23^***^

** P < 0.01.

*** P < 0.001.

Table 3 shows the RRs and *95% CIs* of appendicitis admissions per 10 μg/m^3^ increase in pollutants at various lag days. The results indicated that short-term air pollution exposure was significantly associated with hospitalizations for appendicitis. In the single-day lag models, the most significant estimates all occurred on the current day (lag0), and the effect values were 1.0170 (1.0146–1.0194) for PM_10_, 1.0230 (1.0187–1.0273) for SO_2_, and 1.0648 (1.0509–1.0790) for NO_2_. In moving average exposure models, these three pollutants all maintained a significant positive association with appendicitis admissions from lag01 to lag05. The most significant effects on hospitalizations for appendicitis were all observed at lag01. For every 10 μg/m^3^ increase in pollutants at lag01, the corresponding effects were 1.0179 (1.0129–1.0230) for PM_10_, 1.0236 (1.0184–1.0288) for SO_2_, and 1.0979 (1.0704–1.1262) for NO_2_.

**Table 3:** The effects of appendicitis admissions per 10 μg/m^3^ increase in pollutant concentrations.

**Lag**	**PM_10_**	**SO_2_**	**NO_2_**
0	1.0170 (1.0146–1.0194)^*^	1.0230 (1.0187–1.0273)^*^	1.0648 (1.0509–1.0790)^*^
1	1.0095 (1.0050–1.0141)^*^	1.0133 (1.0086–1.0181)^*^	1.0392 (1.0224–1.0562)^*^
2	1.0021 (0.9976–1.0066)	1.0069 (1.0021–1.0117)^*^	1.0130 (0.9942–1.0321)
3	1.0001 (0.9944–1.0032)	1.0020 (0.9974–1.0067)	0.9926 (0.9728–1.0128)
4	1.0004 (0.9961–1.0048)	0.9987 (0.9941–1.0033)	0.9812 (0.9616–1.0013)
5	1.0006 (0.9962–1.0049)	0.9975 (0.9929–1.0021)	0.9858 (0.9666–1.0054)
01	1.0179 (1.0129–1.0230)^*^	1.0236 (1.0184–1.0288)^*^	1.0979 (1.0704–1.1262)^*^
02	1.0158 (1.0102–1.0213)^*^	1.0235 (1.0176–1.0295)^*^	1.0926 (1.0601–1.1261)^*^
03	1.0137 (1.0076–1.0199)^*^	1.0226 (1.0160–1.0292)^*^	1.0915 (1.0698–1.1136)^*^
04	1.0133 (1.0066–1.0200)^*^	1.0207 (1.0135–1.0280)^*^	1.0797 (1.0431–1.1175)^*^
05	1.0131 (1.0059–1.0204)^*^	1.0192 (1.0113–1.0271)^*^	1.0689 (1.0292–1.1102)^*^

* P < 0.05.

**Figure 2:**
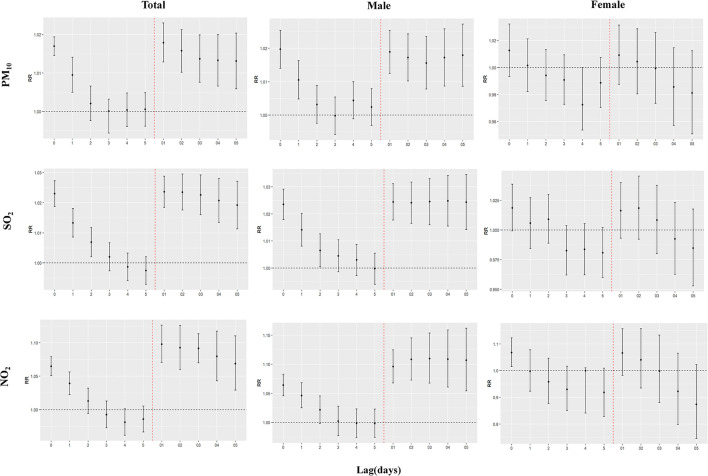
Overall and sex-specific analyses of appendicitis admissions per 10 μg/m^3^ increase in pollutant concentrations.

The overall and sex-specific analyses for appendicitis per 10 μg/m^3^ increase in pollutants are summarized in Figure 2_._ We only found adverse effects of pollutants in the male group, with the strongest effects of 1.0197 (1.0140–1.0254) for PM_10_ at lag0, 1.0248 (1.0155–1.0341) for SO_2_ at lag04 and 1.1097 (1.0674–1.1537) for NO_2_ at lag03. However, no significant effect was found in the female group (Supplementary Table 1).

**Figure 3:**
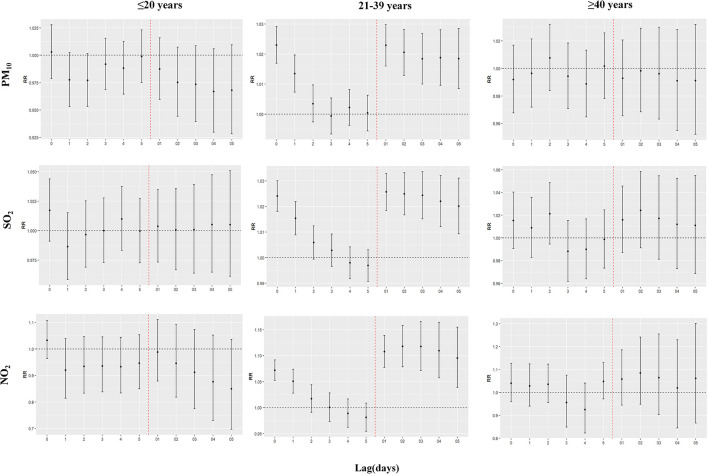
Age-specific analysis of appendicitis admissions per 10 μg/m^3^ increase in pollutant concentrations.

Figure 3 shows the results of the age-specific analysis. Significant adverse effects were observed only in the 21-39 age group, and all occurred at lag0-lag1 and lag01-lag05. The most significant effects of PM_10_, NO_2_, and SO_2_ were 1.0230 (1.0169–1.0292) at lag0, 1.1178 (1.0786–1.1583) at lag02, and 1.0257 (1.0184–1.0329) at lag01, respectively (Supplementary Table 1).

In terms of seasonal stratification, the effects of the cold season seemed to be stronger than those of the warm season, but there was no statistical significance between the groups (Supplementary Table 2).

Table 4 displays the results of appendicitis admissions after adjusting for other pollutants. For SO_2_ and NO_2_, the effects decreased when other pollutants were added to the model, but the associations with appendicitis remained statistically significant in the multipollutant models. For PM_10_, the effect value was still statistically significant when only NO_2_ was adjusted for in the model. However, when only SO_2_ was adjusted for or both NO_2_ and SO_2_ were adjusted for in the model, the association between PM_10_ and appendicitis became statistically non-significant. In addition, when we further adjusted the *df* of the temporal trends (6–9), daily average temperature (3–5) and relative humidity (3–5), the associations between the three pollutants and appendicitis admissions remained statistically significant, indicating that our results were robust (Supplementary Tables 3, 4).

**Table 4:** Results of hospital admissions for appendicitis after adjusting for other pollutants.

**Model**	***RR***	***95% CI***
**PM**_10_		
Single pollutant model	1.0179	1.0129–1.0230^*^
+NO_2_	1.0091	1.0032–1.0149^*^
+SO_2_	1.0044	0.9978–1.0109
+NO_2_+SO_2_	1.0019	0.9952–1.0086
**SO**_2_		
Single pollutant model	1.0236	1.0184–1.0288^*^
+PM_10_	1.0204	1.0133–1.0274^*^
+NO_2_	1.0166	1.0099–1.0232^*^
+PM_10_+NO_2_	1.0154	1.0078–1.0231^*^
**NO**_2_		
Single pollutant model	1.0979	1.0704–1.1262^*^
+PM_10_	1.0697	1.0433–1.0969^*^
+SO_2_	1.0487	1.0197–1.0786^*^
+PM_10_+SO_2_	1.0466	1.0166–1.0775^*^

The most significant effects with multi-day lag (lag01 for PM_10_, SO_2_, and NO_2_) were used for appendicitis admissions.

* P < 0.05.

## Discussion

To date, this may be the first study conducted in mainland China that investigates the impacts of short-term air pollution exposure on appendicitis by using a time series approach. This hospital-based study indicated that short-term exposure to PM_10_, SO_2_, and NO_2_ was significantly associated with daily appendicitis hospitalizations. Following the construction of single- and multipollutant models, the results indicated that gaseous pollutants seemed to have a more pronounced effect on appendicitis than PM_10_. Furthermore, males and people aged 21–39 years seemed to be more susceptible to air pollutants. This study adds to the epidemiological evidence of the effects of air pollution on gastrointestinal disorders.

In 2021, based on the scientific research of the past 15 years, the WHO issued the unprecedentedly strict “Global Air Quality Guidelines” (AQGs), which put forwards greater requirements for atmospheric concentration indicators and determined the 24-h mean concentration standards of PM_10_, SO_2_, and NO_2_ as 45, 40, and 25 μg/m^3^, respectively (31). In our study, the daily average concentrations of PM_10_, SO_2_, and NO_2_ were 117.42, 67.62, and 36.04 μg/m^3^, respectively, which were all considerably higher than the WHO air quality standards. Using the WHO 24-h concentration standards as a guide, during the 1,096 days of the study period, PM_10_ exceeded the standard on 1,017 days, SO_2_ on 492 days, and NO_2_ on 757 days, indicating that the air pollution in Linfen was very serious. According to China's air quality standards, the daily average concentrations of PM_10_ and SO_2_ were both higher than the level I standard (50 μg/m^3^), while NO_2_ concentration was lower than the level I standard (80 μg/m^3^). Furthermore, the average daily concentrations of all three pollutants were below China's level II air quality standards (150 μg/m^3^ for PM_10_ and SO_2_, 80 μg/m^3^ for NO_2_). Nevertheless, the concentration of PM_10_ exceeded China's level II air quality standards on 228 days, SO_2_ on 116 days and NO_2_ on 35 days. Moreover, during the study period, we observed that the daily maximum mean concentration of SO_2_ reached 858 μg/m^3^, which was 21 times the WHO air quality level (40 μg/m^3^), indicating a very serious level of SO_2_ pollution in Linfen city. Linfen's air pollution is so bad that the city has been repeatedly rated as one of the most polluted cities in China by the Ministry of Ecology and Environment. Linfen is rich in coal resources, and the higher concentration of air pollution in winter may be related to coal burning during the heating season. In addition, a large number of companies with high levels of energy consumption and pollution are clustered in the Linfen Basin, which is also the most important reason for Linfen's serious pollution (23). In view of these considerations, it is of great importance to study the impacts of air pollutants on appendicitis admissions in Linfen, a city with serious air pollution in mainland China.

There is relatively little epidemiological evidence of the impacts of air pollution on appendicitis. Studies have mainly been conducted in developed countries, and the results of previous studies are still controversial. Therefore, we collected hospitalization data of appendicitis patients in Linfen city, a city with severe air pollution in mainland China, and conducted this study to look for a possible association with air pollution. Our results found a positive association between air pollutants and appendicitis admissions, which is supported by several published studies. A cross-sectional study in Tunisia compared the impacts of environmental factors on perforated and non-perforated appendicitis and reported that short-term exposure to PM_10_ (2-day lag mean concentration) was significantly associated with perforated appendicitis (RR 1.066, 1.007–1.130) (20). Kaplan et al. performed a case-crossover study involving 5,191 hospitalized patients with appendicitis over the age of 18 in Calgary, Canada, and found that short-term air pollution exposure increased the incidence of appendicitis (15). In addition, the effects were most pronounced for SO_2_ (OR 1.30, 95% CI 1.03–1.63), NO_2_ (OR 1.76, 95% CI 1.20–2.58), and PM_10_ (OR 1.20, 95% CI 1.05–1.38) in summer (July–August) (15). Another case-crossover study also reported a significant adverse effect of NO_2_ on hospitalization for appendicitis in Taiwan, with PM_10_ having a significant effect only during the cold season (below 23°C), while SO_2_ was not found to have a significant effect in either the single- or two-pollutant model in the study (17). Other studies have also shown that PM_10_, SO_2_, or NO_2_ are not associated with appendicitis (18, 19). The reasons for these differences may be the use of different study designs, the selection of the appendicitis population (e.g., ICD codes, perforated, or non-perforated appendicitis), and the concentration and composition of air pollution in different regions. Additionally, studies have demonstrated that other air pollutants (e.g., O_3_ and CO) are associated with the onset of appendicitis. Kaolan et al. showed that the impacts of O_3_ (OR 1.32, 95% CI 1.10–1.57) and CO (OR 1.35, 95% CI 1.01–1.80) in summer were most pronounced with the incidence of appendicitis (15). A study in Taiwan reported that exposure to O_3_ was correlated with the frequency of appendicitis hospitalizations (17). The impact of ambient O_3_ on appendicitis was also confirmed by a multicity case-crossover study. In that study, Kaplan et al. found that higher levels of environmental O_3_ exposure were correlated with perforated appendicitis (16). However, the effects of O_3_ and CO were not analyzed in this study because these data were not available; studies that include these pollutants are urgently needed.

Studies have shown that males are more likely to develop appendicitis than females, with lifetime incidences of 8.6 and 6.7%, respectively (32). In this study, the number of appendicitis hospitalizations was also much higher among males than among females (1,183 vs. 244). In addition, consistent with previous studies, our study showed that males were more likely to be affected by air pollutants than females (16, 32, 33). This finding may be because outdoor work is predominantly performed by males and thus these individuals are more exposed to air pollutants. It is recommended that males be well protected against air pollutants when working outdoors to effectively reduce the number of hospitalizations for appendicitis.

Appendicitis is more likely to occur in younger people (32, 34, 35). In our study, most hospitalized patients with appendicitis were 21–39 years old, and they were more susceptible to pollutants. However, one study reported that people aged over 64 years were more susceptible to NO_2_ than younger adults (age 18–35 years) in terms of appendicitis (15), which was different from our findings. However, the specific reasons for this outcome are still unknown, and further studies are needed to clarify these age-specific effects.

Studies in South Korea, Canada and elsewhere have shown that appendicitis is more likely to occur in summer, which is possibly due to dietary habits (36). During the summer months, people may increase their intake of low-fiber foods and sugar when they go outside, possibly leading to constipation and appendicitis (37). In addition, people may open their windows or go outside on warm days, which further increases their exposure to air pollutants (15). In this study, the impacts of air pollutants seemed to be stronger in the cold season, but the difference between the cold and warm seasons was not statistically significant. The differences in these studies may be due to the climatic characteristics of different regions and different concentrations and components of air pollution. Furthermore, no such studies have been conducted in mainland China, which limits our further in-depth comparative analysis.

In the two- and three- pollutant models, after adding other contaminants to the SO_2_ and NO_2_ models, the effect values decreased somewhat but remained significant. After adding NO_2_ to the PM_10_ model, the effect value was also statistically significant, but when only SO_2_ was adjusted for or both SO_2_ and NO_2_ were adjusted for, the effects became non-significant. This trend suggests that gaseous pollutants may play a greater role in inducing appendicitis than PM_10_. Prevention and control of air pollutants, especially gaseous pollutants, should be strengthened in the future.

The specific mechanism of how air pollutants affect the onset of appendicitis is not yet clear, but the association between the two is somewhat biologically plausible. One study reported that inhaling or ingesting air pollutants may cause an inflammatory response in humans (15). Particles are deposited in the nasopharyngeal chambers, cleared by cilia and swallowed, so they may directly affect the gastrointestinal tract (38). Air pollutants can activate immune cells and lead to cytotoxicity in intestinal epithelial cells (39). Air pollutants can also directly affect the gut microbiota, which may lead to digestive diseases (40). Animal studies have shown that exposing the intestinal tracts of mice to particulate matter alters the colon microbiota structure and increases the levels of interleukin-8 (IL-8) and IL-17 (14). In addition, exposing mice to particulate matter results in changes in gut microbial composition and metabolic processes, which may also contribute to the inflammatory response (12).

Several limitations in this study should not be ignored. First, this study had an ecological design and had some limitations regarding causal inference. Second, the daily cases of hospitalization for appendicitis were only from one hospital in Linfen city, and the conclusions of the study may not be widely generalizable and may not represent other cities and regions with different characteristics. Although only one hospital was included in this study, it was still very representative. In 2021, the number of outpatient and emergency patients in Linfen People's Hospital reached 1,031,200, and the number of discharged patients reached 65,000. This is the largest comprehensive grade A hospital in Linfen with multiple specialties, advanced equipment and a strong technical force. Most patients with appendicitis seek treatment at this hospital. Third, consistent with many published time series studies, this study also used monitoring station data to replace individual exposure data, which inevitably has the problem of exposure measurement errors. In recent years, some more advanced modeling methods, such as land use regression models and satellite inversion, have been applied to assess precise individual exposures, which can improve the spatial resolution of exposure assessment to some extent. In a follow-up study, we will try to apply a more accurate individual exposure assessment method to explore the impacts of air pollutants on appendicitis. Fourth, some individual factors, such as physical condition, occupation, diet, marital status, education and income, may have influenced admissions for appendicitis, but we did not have access to these data (32, 41, 42). Additionally, the onset date of appendicitis may have differed from the date of hospitalization, and studies have reported that up to 20% of patients with appendicitis may have delayed admission to the hospital (43). Further studies that fully consider these limitations are urgently needed.

## Conclusions

In conclusion, our findings indicated that short-term air pollutant (PM_10_, SO_2_, and NO_2_) exposure was significantly correlated with the number of hospitalizations for appendicitis in heavily polluted Linfen city. Males and people aged 21–39 were more susceptible to air pollution. Our study adds to the epidemiological evidence of an association of air pollutants with appendicitis, providing a reference for governments and health authorities to develop targeted air pollution interventions.

## Data availability statement

The raw data supporting the conclusions of this article will be made available by the authors, without undue reservation.

## Ethics statement

Prior to data collection, the Ethics Committee of Shantou University had approved this study. Overall aggregated data were used in our study and no information about individual patient privacy was involved in the analysis.

## Author contributions

YJ and FZ: conceptualization, data curation, investigation, and writing—original draft. ZH and XZ: validation and formal analysis. YC and ZS: conceptualization and writing—review and editing. XS and LL: writing—review and editing and supervision. All authors contributed to the article and approved the submitted version.
