# Supplementary material for: Impacts of short-term air pollution exposure on appendicitis admissions: Evidence from one of the most polluted cities in mainland China
Source: Front Public Health. 2023 Mar 16;11:1144310. doi: 10.3389/fpubh.2023.1144310 (PMC10061118; doi:10.3389/fpubh.2023.1144310)
Supplement: Supplementary file 1 [file Data_Sheet_1.pdf]

Supplementary Material

Impacts of short-term air pollution exposure on appendicitis admissions: Evidence from one of the most polluted cities in mainland China

Yanhu Ji[1,2], Xuefeng Su[3], Fengying Zhang[4], Zepeng Huang[5], Xiaowei Zhang[1,2], Yueliang Chen[1,2], Ziyi Song[1,2], Liping Li[1,2,*]

[1] School of Public Health, Shantou University, 515063, Shantou, China

[2] Injury Prevention Research Center, Shantou University Medical College, 515041, Shantou, China

[3] Linfen People's Hospital, 041000, Linfen, China

[4] China National Environmental Monitoring Center, 100012, Beijing, China

[5] The Second Affiliated Hospital of Shantou University Medical College, 515041, Shantou, China

*** Correspondence:** Liping Li: lpli@stu.edu.cn

Fig. S1 Time series of air pollutants in Linfen, China, from 2016-2018

Table S1 The effects of appendicitis admissions per 10 μg/m^3 increase in pollutant concentrations by sex and age

Table S2 The effects of appendicitis admissions per 10 μg/m^3 increase in pollutant concentrations by season

Table S3 The results of varying the degrees of freedom (6-9 *df*) for time

Table S4 The results of varying the degrees of freedom (3-5 *df*) for mean temperature and relative humidity

Fig. S1 Time series of air pollutants in Linfen, China, from 2016-2018

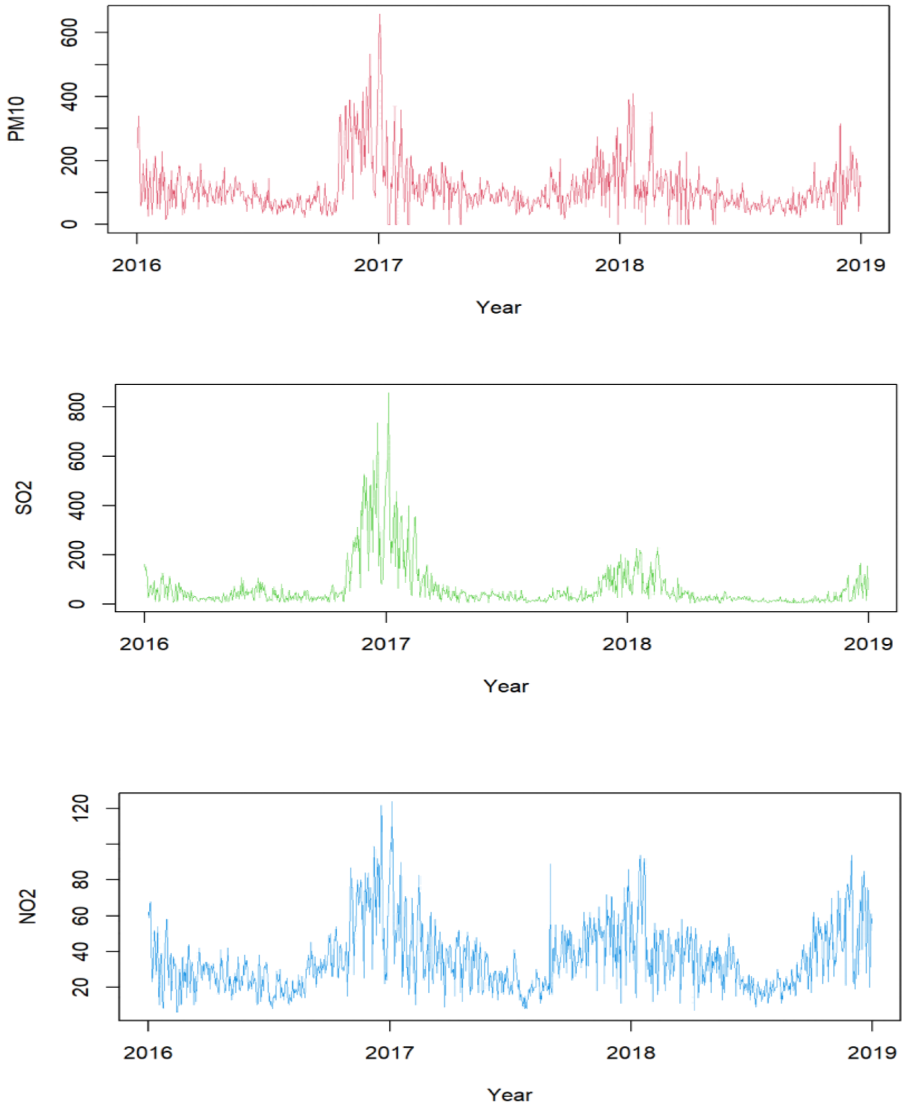

Table S1 The effects of appendicitis admissions per 10 μg/m^3 increase in pollutant concentrations by sex and age

Pollutants	Lag day	Gender		Age		
		Male	Female	≤20 years	21-39 years	≥40 years
PM_{10}	0	1.0197(1.0140-1.0254)*	1.0127(0.9934-1.0322)	1.0029(0.9786-1.0278)	1.0230(1.0169-1.0292)*	0.9920(0.9677-1.0168)
	1	1.0105(1.0048-1.0163)*	1.0016(0.9823-1.0213)	0.9775(0.9532-1.0024)	1.0135(1.0073-1.0197)*	0.9964(0.9719-1.0215)
	2	1.0032(0.9976-1.0089)	0.9942(0.9756-1.0132)	0.9771(0.9533-1.0015)	1.0035(0.9974-1.0097)	1.0076(0.9837-1.0321)
	3	0.9998(0.9942-1.0054)	0.9909(0.9728-1.0095)	0.9919(0.9687-1.0155)	0.9994(0.9934-1.0054)	0.9943(0.9707-1.0184)
	4	1.0044(0.9989-1.0100)	0.9725(0.9537-1.0001)	0.9882(0.9644-1.0126)	1.0022(0.9963-1.0082)	0.9888(0.9648-1.0133)
	5	1.0024(0.9969-1.0079)	0.9887(0.9703-1.0075)	0.9988(0.9751-1.0231)	1.0004(0.9944-1.0063)	1.0017(0.9781-1.0258)
	01	1.0189(1.0125-1.0254)*	1.0092(0.9875-1.0315)	0.9874(0.9597-1.0159)	1.0229(1.0160-1.0298)*	0.9928(0.9656-1.0207)
	02	1.0172(1.0102-1.0243)*	1.0044(0.9805-1.0288)	0.9753(0.9443-1.0074)	1.0206(1.0129-1.0282)*	0.9983(0.9684-1.0292)
	03	1.0156(1.0078-1.0235)*	0.9994(0.9735-1.0260)	0.9735(0.9396-1.0087)	1.0184(1.0100-1.0269)*	0.9961(0.9632-1.0299)
	04	1.0172(1.0087-1.0258)*	0.9856(0.9571-1.0147)	0.9670(0.9297-1.0059)	1.0188(1.0096-1.0281)*	0.9910(0.9549-1.0284)

	05	1.0179(1.0086-1.0272)*	0.9812(0.9509-1.0125)	0.9681(0.9284-1.0096)	1.0185(1.0085-1.0285)*	0.9912(0.9521-1.0320)
	0	1.0235(1.0179-1.0291)*	1.0186(0.9992-1.0385)	1.0172(0.9910-1.0440)	1.0241(1.0181-1.0301)*	1.0153(0.9907-1.0404)
	1	1.0141(1.0081-1.0202)*	1.0057(0.9845-1.0272)	0.9864(0.9584-1.0153)	1.0154(1.0089-1.0219)*	1.0089(0.9828-1.0357)
	2	1.0065(1.0005-1.0126)*	1.0089(0.9888-1.0300)	0.9967(0.9689-1.0253)	1.0059(0.9994-1.0124)	1.0213(0.9946-1.0487)
	3	1.0045(0.9986-1.0104)	0.9826(0.9620-1.0037)	1.0001(0.9731-1.0278)	1.0029(0.9966-1.0092)	0.9882(0.9617-1.0155)
	4	1.0030(0.9972-1.0087)	0.9837(0.9622-1.0053)	1.0100(0.9833-1.0375)	0.9980(0.9918-1.0042)	0.9900(0.9639-1.0168)
SO_2	5	0.9998(0.9940-1.0055)	0.9808(0.9601-1.0020)	0.9998(0.9727-1.0275)	0.9969(0.9907-1.0031)	0.9987(0.9734-1.0247)
	01	1.0244(1.0177-1.0311)*	1.0163(0.9932-1.0400)	1.0037(0.9733-1.0350)	1.0257(1.0184-1.0329)*	1.0159(0.9871-1.0456)
	02	1.0241(1.0165-1.0317)*	1.0184(0.9922-1.0453)	1.0007(0.9668-1.0357)	1.0249(1.0167-1.0332)*	1.0243(0.9913-1.0585)
	03	1.0245(1.0160-1.0330)*	1.0084(0.9801-1.0376)	1.0008(0.9639-1.0391)	1.0244(1.0152-1.0336)*	1.0173(0.9812-1.0548)
	04	1.0248(1.0155-1.0341)*	0.9925(0.9626-1.0233)	1.0053(0.9648-1.0475)	1.0220(1.0121-1.0321)*	1.0120(0.9731-1.0525)
	05	1.0243(1.0142-1.0345)*	0.9848(0.9529-1.0177)	1.0051(0.9613-1.0509)	1.0201(1.0093-1.0311)*	1.0110(0.9688-1.0549)
NO_2	0	1.0641(1.0459-1.0827)*	1.0675(1.0149-1.1228)*	1.0328(0.9633-1.1073)	1.0720(1.0525-1.0919)*	1.0405(0.9598-1.1279)
	1	1.0463(1.0250-1.0680)*	0.9977(0.9233-1.0781)	0.9200(0.8144-1.0394)	1.0508(1.0281-1.0740)*	1.0281(0.9401-1.1243)

	2	1.0216(0.9981-1.0456)	0.9579(0.8770-1.0464)	0.9339(0.8333-1.0467)	1.0173(0.9911-1.0441)	1.0358(0.9551-1.1234)
	3	1.0023(0.9772-1.0280)	0.9296(0.8503-1.0163)	0.9358(0.8377-1.0454)	1.0005(0.9734-1.0284)	0.9559(0.8493-1.0760)
	4	0.9985(0.9740-1.0234)	0.9997(0.8410-1.0102)	0.9331(0.8338-1.0441)	0.9891(0.9622-1.0167)	0.9257(0.8231-1.0411)
	5	0.9983(0.9743-1.0229)	0.9185(0.8278-1.0093)	0.9462(0.8494-1.0539)	0.9812(0.9544-1.0087)	1.0482(0.9717-1.1307)
	01	1.0960(1.0678-1.1248)*	1.0655(0.9816-1.1566)	0.9881(0.8790-1.1107)	1.1079(1.0774-1.1393)*	1.0586(0.9450-1.1858)
	02	1.1085(1.0728-1.1453)*	1.0399(0.9347-1.1569)	0.9457(0.8185-1.0927)	1.1178(1.0786-1.1583)*	1.0852(0.9483-1.2419)
	03	1.1097(1.0674-1.1537)*	0.9984(0.8802-1.1325)	0.9118(0.7743-1.0738)	1.1175(1.0712-1.1659)*	1.0649(0.9039-1.2547)
	04	1.1087(1.0608-1.1587)*	0.9226(0.7988-1.0654)	0.8760(0.7294-1.0521)	1.1096(1.0575-1.1644)*	1.0195(0.8452-1.2298)
	05	1.1068(1.0543-1.1618)*	0.8735(0.7463-1.0225)	0.8495(0.6966-1.0360)	1.0953(1.0387-1.1549)*	1.0615(0.8665-1.3003)

Note. *$P < 0.05$.

Table S2 The effects of appendicitis admissions per 10 μg/m^3 increase in pollutant concentrations by season

Pollutants	Lag day	Season	
		Warm	Cold
PM_{10}	0	1.0355(1.0104-1.0612)*	1.0215(1.0168-1.0262)*
	1	1.0038(0.9803-1.0279)	1.0094(1.0045-1.0144)*
	2	0.9827(0.9600-1.0060)	1.0019(0.9972-1.0066)
	3	0.9865(0.9634-1.0101)	0.9976(0.9930-1.0022)
	4	0.9941(0.9707-1.0181)	0.9981(0.9936-1.0026)
	5	1.0129(0.9894-1.0369)	0.9983(0.9939-1.0028)
	01	1.0281(0.9986-1.0585)	1.0192(1.0139-1.0245)*
	02	1.0127(0.9791-1.0473)	1.0165(1.0106-1.0224)*
	03	1.0034(0.9656-1.0426)	1.0136(1.0071-1.0202)*
	04	0.9995(0.9575-1.0434)	1.0119(1.0048-1.0191)*
	05	1.0082(0.9616-1.0570)	1.0106(1.0029-1.0184)*

SO_2	0	1.1950(1.1465-1.2456)*	1.0200(1.0160-1.0240)*
	1	1.0693(1.0232-1.1174)*	1.0122(1.0077-1.0167)*
	2	1.0165(0.9729-1.0620)	1.0063(1.0018-1.0109)*
	3	1.0044(0.9617-1.0491)	1.0009(0.9965-1.0053)
	4	0.9767(0.9348-1.0204)	0.9984(0.9941-1.0027)
	5	1.0049(0.9621-1.0495)	0.9973(0.9930-1.0016)
	01	1.2298(1.1631-1.3003)*	1.0210(1.0162-1.0259)*
	02	1.2209(1.1429-1.3043)*	1.0214(1.0158-1.0270)*
	03	1.2019(1.1179-1.2921)*	1.0205(1.0142-1.0268)*
	04	1.1701(1.0816-1.2660)*	1.0187(1.0117-1.0257)*
	05	1.1698(1.0743-1.2737)*	1.0170(1.0094-1.0247)*
NO_2	0	1.0879(1.0044-1.1784)*	1.1458(1.1214-1.1707)*
	1	1.1028(1.0179-1.1947)*	1.0693(1.0429-1.0964)*
	2	1.0207(0.9425-1.1054)	1.0186(0.9952-1.0426)

	3	0.9934(0.9186-1.0742)	0.9845(0.9629-1.0066)
	4	0.9298(0.8598-1.0054)	0.9799(0.9591-1.0012)
	5	0.9596(0.8875-1.0375)	0.9848(0.9640-1.0061)
	01	1.1460(1.0377-1.2655)*	1.1518(1.1214-1.1831)*
	02	1.1482(1.0232-1.2885)*	1.1464(1.1108-1.1830)*
	03	1.1351(0.9976-1.2916)	1.1257(1.0857-1.1672)*
	04	1.0780(0.9349-1.2431)	1.1050(1.0614-1.1504)*
	05	1.0492(0.8971-1.2272)	1.0910(1.0442-1.1400)*

Note. *P < 0.05.

Table S3 The results of varying the degrees of freedom (6-9 *dfs*) for time

Pollutants	Time(*df*=6)	Time(*df*=7)	Time(*df*=8)	Time(*df*=9)
PM_{10}	1.0194(1.0142-1.0245)*	1.0179(1.0129-1.0230)*	1.0177(1.0125-1.0229)*	1.0172(1.0121-1.0224)*
SO_2	1.0253(1.0200-1.0307)*	1.0236(1.0184-1.0288)*	1.0234(1.0181-1.0288)*	1.0229(1.0176-1.0283)*
NO_2	1.1019(1.0793-1.1249)*	1.0979(1.0704-1.1262)*	1.0932(1.0704-1.1165)*	1.0955(1.0725-1.1189)*

Note. The maximum effect values with multi-day lag (lag01 for PM_{10}, SO_2 and NO_2) were used for appendicitis hospitalization.*P < 0.05

Table S4 The results of varying the degrees of freedom (3-5 *dfs*) for mean temperature and relative humidity

Pollutants	MT			RH		
	df=3	*df*=4	*df*=5	*df*=3	*df*=4	*df*=5
PM_{10}	1.0179 (1.0129-1.0230)*	1.0178 (1.0128-1.0228)*	1.0179 (1.0128-1.0229)*	1.0179 (1.0129-1.0230)*	1.0179 (1.0129-1.0230)*	1.0179 (1.0129-1.0229)*
SO_2	1.0236 (1.0184-1.0288)*	1.0234 (1.0182-1.0287)*	1.0234 (1.0182-1.0286)*	1.0236 (1.0184-1.0288)*	1.0237 (1.0185-1.0289)*	1.0232 (1.0181-1.0285)*
NO_2	1.0979 (1.0704-1.1262)*	1.0913 (1.0696-1.1133)*	1.0892 (1.0677-1.1112)*	1.0979 (1.0704-1.1262)*	1.0920 (1.0703-1.1142)*	1.0925 (1.0707-1.1147)*

Note. The maximum effect values with multi-day lag (lag01 for PM_{10}, SO_2 and NO_2) were used for appendicitis hospitalization.*$P < 0.05$
